# Effectiveness of educational intervention on quality of life in adults with thalassemia major: A quasi‐experimental study based on PRECEDE model

**DOI:** 10.1002/hsr2.70075

**Published:** 2024-09-23

**Authors:** Laleh Hassani, Niloofar Seyrafi, Sama Mohammadi, Teamur Aghamolaei, Amin Ghanbarnejad, Mohammad Reza Evazi

**Affiliations:** ^1^ Social Determinants in Health Promotion Research Center, Hormozgan Health Research Institute Hormozgan University of Medical Sciences BandarAbbas Iran; ^2^ Faculty of Health Hormozgan University of Medical Sciences BandarAbbas Iran; ^3^ Social Determinants in Health Promotion Research Center, Hormozgan Health Institute Hormozgan University of Medical Sciences BandarAbbas Iran; ^4^ School of Medicine Hormozgan University of Medical Sciences BandarAbbas Iran

**Keywords:** health education, PRECEDE model, quality of life, thalassemia major

## Abstract

**Background and Aim:**

Due to the adverse effects of their disease, patients with thalassemia major face many problems such as a lack of proper growth, enlarged spleen and liver, bone disorders, especially the bones of the head and face. As a result of the long treatment process of this disease, other aspects of their life are also affected. The physical and mental problems in this population of patients will lead to frustration and lower social performance and quality of life. The present study aimed to explore the effect of an educational intervention based on the PRECEDE model on quality of life of adults with thalassemia major in Hormozgan province.

**Methods:**

The present quasi‐experimental study was conducted on 234 adults with major thalassemia who visited the thalassemia medical centers in Hormozgan province in 2022. The participants were selected through a simple randomization from two cities of Hormozgan province (Bandar Abbas as the intervention group and Minab as the control). Each group had an equal number of participants (*n* = 117). The data were collected using a demographic information questionnaire, a researcher‐made questionnaire based on the model constructs and the SF‐12 quality of life questionnaire. The instruments were once administered before the intervention and once again 4 months after the intervention. The educational intervention included 3 sessions a week, each 60 min in length, shared in WhatsApp and Telegram. The data were analyzed in statistical package for the social sciences version 24 using Mann−Whitney *U*‐test, chi‐square test, Wilcoxon's test, and analysis of covariance.

**Findings:**

The results showed that after the educational intervention, the mean scores of knowledge, attitude, self‐efficacy, enabling afctors, reinforcing factors and the mean score of overall quality of life increased significantly in the intervention group compared to the control (*p* < 0.05). The covariance analysis showed when the effect of the pretest score is removed, the mean scores of the model constructs and the quality of life are significantly different in the two groups.

**Conclusion:**

Educational interventions based on the PRECEDE model can help identify factors affecting the quality of life on many aspects in patients with thalassemia major. Thus, these interventions help improve patients' quality of life. The present findings can guide health policy makers and experts use modern educational interventions to develop the educational content to promote a healthy lifestyle and self‐management in certain diseases such as thalassemia major.

## 1 INTRODUCTION

Beta thalassemia is the most common monogenic hereditary disorder on a global scale.^1^ Its most severe form is beta‐thalassemia major, which is associated with severe microcytic hypochromic anemia.^2^ This disease has symptoms of chronic and severe anemia, lack of proper growth, enlarged spleen and liver, bone disorders, especially visible changes in the bones of head and face along with a change in appearance.^3^

The prevalence of this disease in the world is usually higher in the Mediterranean, the areas near the equator and in Africa and Asia.^4^ As reported by the World Health Organization (WHO), 4% (about two million and eight hundred thousand) of the Iranian population have mild or minor thalassemia, and every year 800 people are added to the population of patients with thalassemia major.^5^ In Iran, the highest prevalence of this disease is on the coast of the Caspian Sea and the Persian Gulf. The provinces of Mazandaran, Guilan, Hormozgan, Khuzestan, Kohgiluyeh and Boyer Ahmad, Fars, Bushehr, Sistan and Baluchistan, Kerman, and Isfahan have a high prevalence of thalassemia while Hamadan province has the lowest prevalence.^5^, ^6^ In 2018, Hormozgan province ranked third in Iran for the prevalence of thalassemia major with 2000 thalassemia patients overall, 1500 of whom were diagnosed with thalassemia major.^5^

Today, the existing therapeutic measures and new drugs help improve and control iron overload with strict adherence to the recommended chelation schedule. Thus, these patients' life expectancy has increased. However, suffering from a chronic disease, the mere thought of being different from others, the frequent and long‐term use of medication, changes in appearance, such as bone abnormalities and short stature, can all lower one's self‐image. The treatment, which includes frequent visits to hospital for blood transfusions and treatment for iron accumulation, delayed or absent sexual development and fertility‐related problems, complications such as heart disease, bone disease, diabetes and infection, uncertainty about the future and problems with planning long‐term therapeutic measures affect other aspects of life. They influence the general health, mental health, and quality of life of these patients and their families.^7^, ^8^ For a chronic disease like thalassemia, which requires lifelong management, it is essential to improve the quality of life as part of the treatment. The related literature has shown these patients have a lower quality of life than the general population.^6^ With better blood transfusion methods available, better treatment of iron accumulation and management of complications, supportive care be taken of patients with thalassemia, and now they live an almost ordinary life with a good quality of life.^9^

The health‐related quality of life is generally thought of as a multidimensional construct representing a patient's perceived impact of the disease and treatment on physical, mental and social well‐being.^8^ The quality of life was defined by WHO as the perception of oneself and one's position in life, within the framework of cultural and value systems, in relation to goals and expectations, norms and concerns.^8^

Several studies have explored the quality of life of patients with thalassemia.^10^, ^11^, ^12^ Recognition of the correlates of the quality of life in patients with thalassemia has direct implications for the design and implementation of clinical interventions, counseling and support to improve the therapeutic outcomes and quality of life in these patients. In this study, the PRECEDE model was used to explore factors affecting the quality of life in patients with thalassemia major. This model was developed in 1970 by Lawrence Green et al. This comprehensive model provides for planning to empower people to actively engage in society to improve their quality of life.^11^ It involves four stages, including: (1) social diagnosis, (2) epidemiological diagnosis, behavioral and environmental diagnosis, (3) educational and ecological diagnosis (exploring enabling and reinforcing factors affecting the process of initiating and maintaining behavior change), (4) administrative and policy diagnosis. According to Green and Kreuter, behavioral factors, as determinants of a particular behavior, can be classified as predisposing, enabling, and reinforcing factors. Predisposing factors are predictors of behavior that include knowledge, attitudes, beliefs, values, perceptions, existing skills, and self‐efficacy. Enabling factors are predictors of behavioral and environmental changes, including accessibility, availability, rules, and policies. Reinforcing factors are factors that follow a behavior and provide ongoing rewards or motivations and include social support and important others.^12^

Planning to improve the health state of patients requires sufficient information about the different dimensions of the patients' quality of life. It is possible to use the PRECEDE model to explore the individual, environmental and social factors affecting the quality of life of these patients. This model is flexible, measurable, and committed to the principle of participation and has a certain procedural structure. The PRECEDE model provides a framework by which predisposing factors (knowledge, attitudes, perceptions, beliefs, etc.), reinforcing factors (influence of others, family, peers, health workers, etc.) and enabling factors (availability of resources, skills, etc.) are determined as factors influencing behavior in educational decision‐making. In fact, the most useful application of this model is to explain the factors related to behavior. Thus, the present study aimed to explore the effect of an educational intervention based on the PRECEDE model on the quality of life of adults with thalassemia major. The following hypotheses were formulated:

1. There is a significant difference between the mean scores of predisposing factors (knowledge, attitude and self‐efficacy) in adults with thalassemia in the intervention and control groups, before and after the educational intervention.
2. There is a significant difference between the mean scores of enabling factors in adults with thalassemia in the intervention and control groups, before and after the educational intervention.
3. There is a significant difference between the mean scores of reinforcing factors in adults with thalassemia in the intervention and control groups, before and after the educational intervention.
4. There is a significant difference between the mean scores of quality of life in adults with thalassemia in the intervention and control groups, before and after the educational intervention.

## 2 METHODS

### 2.1 Research design and population

The present quasi‐experimental study was conducted with a control and an intervention group. The research population included all adults with thalassemia major visiting thalassemia treatment centers in Hormozgan province.

### 2.2 Sample size and sampling procedure

According to previous studies,^13^ the standard deviation was equal to 33.38. With a standard error of 5%, the test power of 80%, the sample size was calculated using the following formula:

$$n=\frac{2\times {\left(Z_{1-\frac{\alpha }{2}}+z_{1-\beta }\right)}^{2}\sigma^{2}}{d^{2}}≅107$$

With a 10% attrition rate, the total sample size was estimated at 234 (117 in the intervention group and 117 in the control). For sampling, at first, a list of thalassemia centers in Hormozgan province was prepared and simple randomization was used to select one center as the intervention group in Bandar Abbas and one as the control in Minab. In the next stage, having gained the required permission from the health centers, the required sample size was selected among patients with thalassemia major based on their medical records using a simple randomization. The inclusion criteria were: 18 years of age or older, consent to participate in the study, and no major problem in physical and mental health. (It should be noted that mental disorders meant not conforming to the mental health, in which case the person is not qualified to participate in the study and provide correct information. Mental disorders in our study are problems such as dementia, drug abuse, suicidal thoughts, and so forth. As for physical health, it is noteworthy that the occurrence of a physical disease in excess of the complications of the disease such as diabetes, kidney and liver disease, and so forth, is the reason for unwillingness to participate or continue with the study. An example is damage to the spine that disrupts one's movement or other physical problems that are not caused by the thalassemia). The exclusion criteria were: absence for more than one training session and having a major problem in physical and mental health.

### 2.3 Data collection

The data collection was online due to the coincidence of the study with the outbreak of COVID‐19 in Iran. To protect the participants' health, the data collection and educational intervention were held online. The questionnaire hyperlink was created and sent to all participants on WhatsApp and Telegram. Before the intervention, the questionnaire was sent to the participants in both research groups. The results of the pretest were analyzed to explore the educational content, educational methods and the number of educational sessions required. Four months after the educational intervention, the data were collected again in both research groups.

### 2.4 Instrumentation

The data was collected using the following questionnaires:

1. Demographic information questionnaire: including age, sex, employment, education level, marital status, socioeconomic status (SES).
2. Standard questionnaire of quality of life (SF‐12): This questionnaire is an abridged form of the questionnaire of quality of life with 36 items developed by War, Keller and Kasinski to measure the physical and mental quality of life. This questionnaire includes 12 questions and eight subscales: physical functioning, role‐physical, bodily pain, general health, vitality, social functioning, role‐emotional, and mental health. The minimum and maximum score that can be obtained is 12 and 48, and a lower score indicates a lower quality of life and vice versa. This questionnaire was translated into Persian by Montazeri et al.^14^ in Iran, and its validity and reliability were estimated at 0.72−0.83 for physical and mental functioning.
3. Researcher‐made adaptation of PRECEDE model: This questionnaire contains 47 questions based on the constituent constructs of the PRECEDE model, including knowledge (12 questions), attitude (12 questions), self‐efficacy (7 questions), enabling factors (8 questions) and reinforcing factors (8 questions). The questionnaire on knowledge of thalassemia, the consequences of the disease, its impact on the quality of life and ways to improve the quality of life included 12 questions to be rated as True/False/Don't Know. A True answer received a score of 1, while a No or Don't Know answer received zero. In this section, the minimum and maximum scores that can be obtained were 0 and 12, respectively.

The questionnaire assessing attitude towards the consequences of thalassemia and improving the quality of life included 12 questions rated on a 5‐point Likert scale (5 = strongly agree, 1 = strongly disagree). In this section, the minimum and maximum scores that could be obtained were 12 and 60, respectively.

The questionnaire assessing the perceived self‐efficacy in improving one's quality of life included seven items rated on a 5‐point Likert scale (5 = always, 1 = never). In this section, the minimum and maximum scores that could be obtained were 7 and 35, respectively.

The questionnaire on enabling factors included eight questions to rate improvement in quality of life, availability of resources and access to resources. These items were scored on a 5‐point Likert scale (5 = always, 1 = never). In this section, the minimum and maximum scores that could be obtained were 8 and 40, respectively.

The questionnaire on reinforcing factors included eight questions to rate family and friends' support and one's own positive feeling of showing behaviors to improve the quality of life. This questionnaire was rated on a 5‐point Likert scale (5 = always, 1 = never). In this section, the minimum and maximum scores that could be obtained were 8 and 40, respectively.

To test the content and face validity of the questionnaire, after developing the items, the questionnaire was submitted to a panel of 10 health education and health promotion experts. Their comments were used to revise the questionnaire. To test the reliability, a pilot test was run and the questionnaire was distributed twice (at a 2‐week interval) among 30 individuals who did not belong to the intervention and control groups. Finally, the reliability of the questionnaire was tested using Cronbach's alpha test of internal consistency. The estimated values for all measurement instruments were above 0.85. The reliability of the model constructs was estimated at 0.87 for knowledge, 0.91 for attitude, 0.83 for self‐efficacy, 0.86 for enabling factors, and 0.89 for reinforcing factors.

### 2.5 Educational intervention

In the present study, after an initial needs assessment and the pretest results, material development based on the needs of the target population and using reliable scientific sources (such as the National Family Self‐Care Guide,^3^ the Education and Health Promotion Office of the Ministry of Health of Iran), the content was developed according to the learner's goals and educational needs. These included: thalassemia and its symptoms, diagnosis of thalassemia major, symptoms, therapeutic measures, iron removal drugs, educational videos on Desferal injection with a special pump, home care, nutritional recommendations in thalassemia, effect of physical activities and sports training, correcting misconceptions about thalassemia, use of subcutaneous iron chelator (including timely visits to receive subcutaneous desferal and adherence to the schedule set by the thalassemia center for blood transfusion and reception of chelator), patient support to adhere to medication by family and health staff (including patient encouragement by family to pay regular visits to receive subcutaneous chelator and patient encouragement by the health staff to pay regular visits to receive subcutaneous chelator) and patient support by family and health staff to improve psychological problems (including patient encouragement by family, friends and health staff to show self‐management behaviors and construction of appropriate educational spaces to provide consultation for patients with major thalassemia by the health staff).

The educational intervention for the experimental group included three educational sessions (each 60 min long), weekly for the intervention groups (six groups of 14−18, a total of 18 educational sessions) shared in WhatsApp and Telegram as lectures by the researcher and an expert holding a master's degree of clinical psychology, Q&As, photo and video screenings). After the end of the training sessions, the necessary materials were provided to the subjects in the form of educational pamphlets. In this study, the educational planning was based on an active learning approach and during the educational intervention, the participants were actively engaged in the educational program. The details of the educational intervention are presented in Table 1.

**Table 1:** Detailed program and training sessions.

Session	Effective factor	Educational intervention
Session 1	Predisposing factor	Increasing awareness of thalassemia major, ways of transmission, symptoms of the disease, therapeutic measures, changing attitudes about healthy lifestyle by adopting self‐management behaviors towards thalassemia major, empowering patients through self‐management behaviors such as regular blood transfusions to improve people's lifestyle and general health.
Session 2	Enabling factor	Teaching life skills by a clinical psychologist, recommendations to follow a nutritional diet, training on individual skills in controlling thalassemia major, correct use of iron removal drugs and desferal pump, training on care during injection by the project manager.
Session 3	Reinforcing factor	Requiring the presence of a family member in the training session, training on home‐care, support and verbal encouragement to continue healthy behavior, teaching life skills by the project manager and clinical psychologist.

After the training sessions, every week training messages were sent to the intervention group about how to improve the quality of life. Also, a follow‐up session was held for these participants on a monthly basis. The follow‐up meetings were set up at the end of each month by the present researcher, and a total number of four follow‐up meetings were made during 4 months. In these meetings, to remind and review the previously taught material, the summary of the training content was sent to the participants through a short training video. During the month, the participants seized the opportunity to ask their questions and also share their experiences with each other. Finally, 4 months after the educational intervention, questionnaires were completed again by both intervention and control groups.

### 2.6 Data analysis

The data were analyzed in SPSS 24, using Chi‐square, Mann−Whitney *U*‐test and Wilcoxon test to compare the two groups in terms of the demographic variables. Analysis of covariance was used to test the relationship between the mean scores of the PRECEDE model and the mean scores of the quality of life. *p* < 0.05 was considered as the significance level.

### 2.7 Ethical considerations

This research was approved by the ethics committee of Bandar Abbas University of Medical Sciences (#IR‐HUMS.IREC.1399.148). In advance to the study, the participants were informed about the procedure and purpose of study and were assured of the confidentiality of the information they provided. An informed written consent was obtained from the participants. To comply with ethical rules, at the end of the study, a training session was held for the control group.

## 3 RESULTS

The minimum age of the participants in both groups was 18 years; their maximum age was 53 years in the intervention group and 40 years in the control. The mean and standard deviation of the participants' age was 26.92 ± 6.23 in the intervention group and 25.38 ± 5.14 in the control. Most participants in the study were female (56.4%), held a diploma and were unemployed (Table 2).

**Table 2:** Distribution of demographic variables in the research groups.

Variable	level	Experimental group *f* (%)	Control group *f* (%)	*p* Value
Sex	Male	48 (41)	54 (46)	
	Female	69 (59)	63 (54)	0.42
Marital status	Single	99 (85)	96 (82)	
	Married	18 (15)	21 (18)	
Education level	Illiterate	3 (3)	1 (1)	0.59
	<Diploma	28 (32)	43 (36)	
	Diploma	42 (36)	63 (54)	0.0001
	University degree	34 (29)	9 (10)	
Employment	Unemployed	76 (65)	81 (69)	
	employed	22 (19)	24 (21)	0.26
	School student	6 (5)	1 (1)	
	University student	13 (11)	11 (9)	
Socioeconomic status	Low	7 (6)	14 (12)	
	Moderate	79 (68)	85 (73)	
	High	15 (13)	15 (13)	0.01
	Very high	16 (14)	3 (2)	

The information in Table 2 shows that before the intervention, there was no statistically significant difference between the two groups in terms of sex, marital status, and employment (*p* < 0.05).

The findings summarized in Table 3 show a statistically significant difference between the mean scores of knowledge, attitude, self‐efficacy of enabling and reinforcing factors in the two groups before the educational intervention (*p* < 0.05). In terms of the total quality of life score, there was no statistically significant difference between the two groups before the intervention (*p* = 0.282). The results of pairwise comparisons by Wilcoxon test in the intervention group showed a significant increase in the mean scores of knowledge, attitude, self‐efficacy, enabling factors, reinforcing factors and overall quality of life before and after the intervention (*p* < 0.001).

**Table 3:** Between‐group comparison of the mean scores of PRECEDE model constructs before and 4 months after intervention.

PRECEDE constructs	Experimental group		Control group		Mann‐Whitney U‐test
	Mean	Standard deviation	Mean	Standard deviation	
Knowledge					
before	7.51	1.45	7.04	1.26	*p* = 0.004
after	10.38	1.19	7.21	1.17	*p* < 0.001
Wilcoxon's test	*p* < 0.001		*p* = 0.136		
Attitude					
before	41.35	7.34	34.34	5.11	*p* < 0.001
after	43.24	7.79	34.48	5.19	*p* < 0.001
Wilcoxon's test	*p* < 0.001		*p* = 0.120		
Self‐efficacy					
before	21.95	3.38	19.21	2.79	*p* < 0.001
after	24.51	2.94	19.26	2.65	*p* < 0.001
Wilcoxon's test	*p* < 0.001		*p* = 0.456		
Enabling factors					
before	22.86	3.89	21.3	2.37	*p* = 0.001
after	26.84	3.66	21.01	2.36	*p* < 0.001
Wilcoxon's test	*p* < 0.001		*p* = 0.625		
Reinforcing factors					
before	23.61	4.56	21.48	3.27	*p* < 0.001
after	28.19	4.84	20.86	3.35	*p* < 0.001
Wilcoxon's test	*p* < 0.001		*p* < 0.001		
Quality of life					
before	27.4	3.89	26.8	2.53	*p* = 0.282
after	31.47	3.45	25.47	2.51	*p* < 0.001
Wilcoxon's test	*p* < 0.001		*p* < 0.001		

To compare the mean scores of the model constructs and the quality of life after controlling the effect of the pretest in the two research groups, analysis of covariance test was used. The results showed a statistically significant difference between the mean scores of the PRECDE model constructs and the quality of life after removing the pretest effect in the two groups. The mean scores of the model constructs and the quality of life in the intervention group were higher than the control. The highest estimated adjusted value was that of the reinforcing factors, explaining about 10% of variation in the quality of life as the dependent variable of (Table 4).

**Table 4:** The results of covariance analysis of model constructs and quality of life in two groups after the intervention.

Source of change	Sum of squares	*df*	Square means	*F* Value	*p* Value
Knowledge	492.82	1	492.82	505.27	*p* < 0.0001
Attitude	259.37	1	259.37	23.49	*p* < 0.0001
Self‐efficacy	468.47	1	468.47	253.17	*p* < 0.0001
Enabling factors	1043.36	1	1043.36	338.75	*p* < 0.0001
Reinforcing factors	1582.15	1	1582.15	368.69	*p* < 0.0001
Quality of life	1805.7	1	1805.7	474.84	*p* < 0.0001

## 4 DISCUSSION

This study aimed to explore the effect of an educational intervention based on the PRECEDE model on improving the quality of life in adults with thalassemia major in Iran. The results showed that the educational intervention increased predisposing factors (knowledge, attitude, and self‐efficacy) in the intervention group compared to the control group. These findings are in consistent with previous studies conducted by Moradi et al.^15^ Dehdari et al.^16^ Wang et al.^17^ Naderian et al.^18^ Sabzmekan et al.^19^ Metin et al.^20^ Dushangir et al.^21^ and Bazpour et al.^22^ Naderian et al.^18^ investigated the quality of life in the elderly in Zahedan and found a significant difference between the mean scores of predisposing factors (knowledge and attitude) of the intervention and control groups after the intervention. Wang et al.^17^ also showed that the mean scores of knowledge and attitude increased significantly after the intervention in the intervention group in terms of the effect of education based on the PRECEDE model on the quality of life in elderly patients with chronic heart failure. According to Bandura, self‐efficacy is confidence in one's abilities to fulfill a task successfully.^23^ Self‐efficacy is a mediating factor between knowledge and behavior, and is the belief in one's ability to show a certain expected behavior. Only knowing what to do and why to do so is not enough to lead to a certain behavior. We are expected to perceive ourselves capable of showing that specific behavior.^24^ In another study by Targah et al.^25^ the educational intervention led to an increase in participants' self‐efficacy and empowerment to improve self‐care behaviors related to thalassemia major. The results of Raisi et al.'s study^26^ also showed that a group‐based mindfulness stress reduction program managed to increase pain self‐efficacy in patients with thalassemia major. Increasing self‐efficacy and management can help improve chronic pain issues. One way to improve self‐efficacy in patients with thalassemia major is to empower them through self‐care training, verbal persuasion to increase self‐efficacy to deal with the adverse effects of the disease, self‐management of thalassemia, living a correct lifestyle and successfully function in daily life.^27^

The present findings concerning the enabling factors revealed a statistically significant difference between the two groups after the intervention. The mean score of enabling factors in the intervention group were higher than the control group. Also, the intervention increased the enabling factors in the participants of the intervention group. This finding is similar with findings from other studies conducted by Bazpour et al.^22^ Ghaibizadeh et al.^28^ Wang et al.^17^ and Naderian et al.^18^ In the present study, the possibility of using educational resources about thalassemia major, access to educational resources through mass media (radio, television, etc.) or the health staff were considered as the enabling factors. The findings reported by Bazpour et al.^22^ about adolescents with beta‐thalassemia major showed that the educational intervention based on the PROCEDE model positively affected the model constructs and a healthy lifestyle factors. Educational programs can improve adolescents' knowledge of and attitude toward thalassemia major and a healthy lifestyle. The research findings by Abedi et al.^29^ and Mousavi et al.^30^ are inconsistent with the findings in the present study. The results of Mousavi et al.'s study^30^ on the effect of training based on the PRECDE model on hemodialysis patients' self‐care behavior showed no significant difference between the mean scores of enabling factors in the intervention group, immediately and 3 months after the intervention. The reason for the divergent findings with Mousavi et al.'s study^30^ can be explained by the different population and sample size, and different lengths of the educational intervention process. The research by Abedi et al.^29^ on patients with hypertension showed that after the educational intervention, the mean scores of all the PRECEDE model constructs except for the enabling factors were significantly different between two groups. Abedi et al.^29^ argued the reason for the discrepancy of their findings with other studies was the initiation of the national blood pressure control plan by the Ministry of Health and the provision of educational services and self‐care behaviors on multiple media.

The present study also showed that the educational intervention in the intervention group led to an increase in the mean scores of reinforcing factors compared to the control group. This finding is in line with the results of studies conducted by Abedi et al.^29^ Naderian et al.^18^ Dehdari et al.^16^ Wang et al.^17^ Sabzmekan et al.^19^ Mousavi et al.^30^ and Afkari et al.^31^ Yet, this finding was different from Metin et al.'s study,^20^ in which the support provided by family, friends, colleagues and health staff, and feeling satisfied with physical exercises were considered as the reinforcing factors to improve the quality of life in adults with thalassemia major. In their study on the quality of life of patients after a coronary artery bypass surgery, Dehdari et al.^16^ followed up the patients twice a week on phone and provided the necessary encouragement to reduce stress. In this study, patients' own positive experiences were considered a source of reinforcement. These researchers held a discussion session with Q&As for families and an educational booklet to increase family support for the patient and help him/her control anxiety. In a study by Metin et al.^20^ on the elderly population in Tehran, the relationship between the reinforcing factors and the quality of life was not significant before and after the intervention in the intervention group.

Finally, a statistically significant difference was found between the mean scores of quality of life in the intervention and control groups after the educational intervention. The mean score of the quality of life were higher in the intervention group than the control. This finding is consistent with the results of studies conducted by Naderian et al.^18^ Dehdari et al.^16^ Afkari et al.^31^ Sabzmekan et al.^19^ and Bazpour et al.^22^ Similar to the present study, in another study by Naderian et al.^18^ on the elderly, the mean score of quality of life decreased in the control group after the educational intervention in contrast to the intervention group. But the mean score of quality of life increased significantly in the intervention group. The findings of Dehdari et al.'s study^16^ showed a significant difference in the dimensions of physical functioning, role‐physical, bodily pain, general health, vitality, social functioning, role‐emotional, mental health, quality of life of patients in the intervention group compared to the control. These differenced emerged after the implementation of the intervention. In another study conducted by Rafiei et al.^32^ the educational intervention could not increase the quality of life in patients with thalassemia and failed to positively affect the different dimensions of quality of life. These researchers attributed this lack of significance to the chronicity of thalassemia disease, the short duration of interventions, the multicausality of quality‐of‐life problems, the negative effect of the disease on different aspects of one's life, and long‐term and continuous interventions to check the effectiveness of interventions. Thus, one goal of care‐taking in these patients should be to improve their quality of life through continuous educational interventions using up‐to‐date educational methods (group discussion, Q&A, and educational videos).

Among the limitations of the present study are: (1) failure to follow up subjects longer to check the consistency of the healthy behavior, (2) a mere focus on the PRECEDE model constructs, (3) the data were collected online and only literate people with internet access were able to participate in the study and the findings may be prone to selection bias, (4) there are more participants with university degrees and with very high SES in the experimental group versus the control group and this might have impacted the results.

Thus, future researchers are recommended to develop qualitative studies to diagnose factors affecting the quality of life of these patients based on the PRECEDE model and also explore these factors using other health education theories or models.

## 5 CONCLUSION

The present findings showed the effectiveness of the PRECEDE model to improve the quality of life in patients with thalassemia major. Effective educational interventions through the improvement of intermediary variables such as knowledge, attitudes and self‐efficacy lead to change and improvement in one or more aspects of self‐care behavior, including changes in lifestyle, self‐care in controlling risks and complications induced by the disease. Also, attention to self‐efficacy and its increase is an important predictor of adopting healthy behaviors by patients with thalassemia major. It is, thus, essential to implement the well‐developed educational programs and hold group training courses for this population of patients to change their lifestyle and empower them to promote health‐seeking behaviors. Considering the positive effectiveness of the PRECEDE model in improving the quality of life of patients with thalassemia major, the findings can be used to enrich theory‐based interventional strategies to develop and change health‐related behaviors.

## AUTHOR CONTRIBUTIONS

**Laleh Hassani**: Conceptualization; methodology; supervision; funding acquisition; writing—review and editing. **Niloofar Seyrafi**: Data curation; project administration; writing—original draft. **Sama Mohammadi**: Conceptualization; data curation; writing—review and editing; writing—original draft; project administration. **Teamur Aghamolaei**: Conceptualization; supervision; project administration; writing—review and editing. **Amin Ghanbarnejad**: Software; methodology; project administration. **Mohammad Reza Evazi**: Writing—review and editing; project administration.

## CONFLICT OF INTERESTS STATEMENT

The authors declare no conflict of interest.

## ETHICS STATEMENT

All methods were performed in accordance with the Declaration of Helsinki. Ethical approval was received for this study from the Ethics Committee of the Hormozgan University of Medical Sciences (IR.HUMS.REC.1399.148). Written informed consent was obtained from individuals who participated in this study. The authors confirm that all methods were performed in accordance with the relevant guidelines and regulations.

## TRANSPARENCY STATEMENT

The lead author Sama Mohammadi affirms that this manuscript is an honest, accurate, and transparent account of the study being reported; that no important aspects of the study have been omitted; and that any discrepancies from the study as planned (and, if relevant, registered) have been explained.

## Data Availability

All authors have read and approved the final version of the manuscript. Sama Mohammadi had full access to all of the data in this study and takes complete responsibility for the integrity of the data and the accuracy of the data analysis.

## 1

Demographic and background information:

1. Age:……… years
2. Sex: (a) female (b) male
3. Marital status: (a) single (b) married
4. Education level: (a) illiterate (b) elementary school (c) junior high school (d) high school (diploma) (e) university degree
5. Occupation: (a) schooler (b) university student (c) employed (d) unemployed
6. Family income:……. rials
7. Type of iron chelation you use: (a) subcutaneous (b) oral (c) mixed
8. Frequency of weekly iron chelation shots:…….
9. The number of other family members afflicted with thalassemia:………

Short‐Form Health Survey (SF‐12):

1. How do you perceive your current state of health?

excellent  very good  good  average  poor

Questions 2 and 3 enquire about your daily activities. Has your current state of health limited your daily activities? If yes, you will say to what extent.

**Table:**

activity	Limited to a great extent	Limited to some extent	Not limited at all
2. Common activities such as moving a table, vacuum cleaning the house, and doing light exercises			
3. Climbing consecutive stairs			

Within the past 4 weeks, did you face any problem due to the poor state of health?

**Table:**

	Yes	No
4. Did you have to do your daily activities less than you always did?		
5. Did you face any limitation in specific activities or others you often do?		

Within the past 4 weeks, did you face any problem in your daily activities during your spiritual health?

**Table:**

	Yes	No
6. Did you do your activities less than you wanted?		
7. Did you do your activities with less precision and care you always had?		

8. Within the past 4 weeks, to what extent did you suffer in doing daily activities?

not at all  a little  to an average extent  to a large extent  extremely

The next question asks about how you felt within the past 4 weeks or simply about your emotional experience. Please mark the answer that first comes to your mind.

**Table:**

Question	Always	Often	Usually	Sometimes	Never
9. How often did you feel calm and safe?					
10. How often did you feel full of energy?					
11. How often did you feel sad or depressed?					

12. Within the past 4 weeks, to what extent did your physical and spiritual problems affect your social affairs? (e.g., meeting friends, relatives, etc.)

always  often  sometimes  rarely  never

Knowledge section:

**Table:**

Statement	True	False	Don't know
1. Thalassemia is not transferred genetically.			
2. Subcutaneous iron chelator (Desferal) helps remove iron overload from body.			
3. The major side effect of recurrent use of subcutaneous iron chelator is the increased iron storage in body.			
4. Drinking tea or coffee after the meal helps remove iron overload from body.			
5. Consuming foods such as meat and liver increase iron overload in body.			
6. The risk of infection in those afflicted with thalassemia is higher than the healthy.			
7. Continuous diarrhea and stomachache are among the side effects of subcutaneous iron chelator (Desferal).			
8. Consuming dairy products (milk, yoghurt, cheese, etc.) increase iron in body.			
9. Irregular blood transfusion and chelator change flat bones like skull roof bone, sternum and ribs			
10. Heart dysfunction is one side effect of iron overload in body.			
11. Atrophy of gums and jaw bone is one complication of thalassemia.			
12. Bone marrow transplantation is one definitive treatment of thalassemia.			

Attitude section:

Read the following statements carefully and show whether you agree or disagree.

**Table:**

Statement	Strongly agree	Agree	Undecided	Disagree	Strongly disagree
1. I don't believe in following the recommendations of controlled nutrition as I have no symptoms of the disease.					
2. It upsets me to think about the consequences of my disease.					
3. I think subcutaneous chelator is more painful than other subcutaneous drugs.					
4. I think regular blood transfusion helps reduce symptoms of fatigue and weakness.					
5. I think my facial bone changes repels some of my friends.					
6. I believe one way to treat this disease is blood transfusion.					
7. Despite the pain caused by subcutaneous chelator, I believe it can control the side effects of the disease.					
8. I believe my disease does not limit my physical activities.					
9. I believe following the recommendations of medical team helps better tolerate the long‐term treatment.					
10. I think recurrent visits for examination and medical interventions helps improve the quality of life.					
11. Regular visits to doctors and medical team helps create peace of mind.					
12. I believe going on a diet with limited meat and vegetables replete with iron helps reduce the side effects of the disease.					

Enablers section (existence of sources, availability of sources):

**Table:**

Enabler	Always	Often	Sometimes	Rarely	Never
1. Training courses on how to promote health and quality of life are available to me.					
2. During the training course, instructions on how to promote health and quality of life were provided to me.					
3. I have the required skill to lower stress and anxiety.					
4. I have the required skill to communicate with others especially my family members.					
5. I have the required skill to take my medicine.					
6. I have the required skill to adhere to personal health especially oral/dental health.					
7. Due to the limited available sources of Desferal Infusion Pumps in thalassemia association of Iran, it is hard for me to access pumps.					
8. Due to the commuting problem, I find it hard to receive blood on time.					

Reinforcement section (reward and punishment):

**Table:**

Reinforcement factor	Always	Often	Sometimes	Rarely	Never
1. My family encourages me to be socially active.					
2. My family supports me in using chelators correctly.					
3. If feeling disappointed, I am cheered up by friends.					
4. If I need advice, the medical team encourages me to get consultation services.					
5. My family encourages me to go for blood transfusion on time.					
6. My family supports me in following oral/dental health advice.					
7. I prefer the good feeling of physical activity and exercises to the physical exhaustion afterwards.					
8. As the thalassemia association does not support patients adequately, I am not motivated to join this association and cooperate with it.					

Self‐efficacy section:

**Table:**

Self‐efficacy	Always	Often	Sometimes	Rarely	Never
1. Despite the disease, I do not stop being socially active.					
2. I stop eating foods that cannot be consumed together with iron chelators.					
3. I continue to take a good care of myself and do not lose hope.					
4. If I need advice, the medical team invites me to visit for consultation.					
5. For blood transfusion, I pay visits even if I have to companion.					
6. I adhere to physical exercises even though I am exhausted, and try not to stop them and instead work out less.					
7. I try to cooperate with the association of thalassemia to see friends and other patients even if I lack the required financial and spiritual support.					
